# Association of enlarged perivascular spaces and anticoagulant-related intracranial hemorrhage

**DOI:** 10.1212/WNL.0000000000010788

**Published:** 2020-10-20

**Authors:** Jonathan G. Best, Carmen Barbato, Gareth Ambler, Houwei Du, Gargi Banerjee, Duncan Wilson, Clare Shakeshaft, Hannah Cohen, Tarek A. Yousry, Rustam Al-Shahi Salman, Gregory Y.H. Lip, Henry Houlden, Martin M. Brown, Keith W. Muir, Hans Rolf Jäger, David J. Werring, on behalf of the Clinical Relevance of Microbleeds in Stroke (CROMIS-2) collaborators, Parry- Jones Adrian, Chris Patterson, Christopher Price, Abduelbaset Elmarimi, Anthea Parry, Arumug Nallasivam, Azlisham Mohd Nor, Bernard Esis, Fabio Nascimento, David Bruce, Biju Bhaskaran, Christine Roffe, Claire Cullen, Clare Holmes, David Cohen, Claire Cullen, Claire Cullen, Claire Cullen, Claire Cullen, Claire Cullen, Claire Cullen, Claire Cullen, Claire Cullen, Claire Cullen, Claire Cullen, Claire Cullen, Claire Cullen, David Hargroves, David Mangion, Dinesh Chadha, Djamil Vahidassr, Dulka Manawadu, Elio Giallombardo, Elizabeth Warburton, Enrico Flossman, Gunaratam Gunathilagan, Harald Proschel, Hedley Emsley, Ijaz Anwar, Ilse Burger, James Okwera, Janet Putterill, Janice O'Connell, John Bamford, John Corrigan, Jon Scott, Jonathan Birns, Karen Kee, Kari Saastamoinen, Kath Pasco, Krishna Dani, Lakshmanan Sekaran, Lillian Choy, Liz Iveson, Maam Mamun, Mahmud Sajid, Martin Cooper, Matthew Burn, Matthew Smith, Michael Power, Michelle Davis, Nigel Smyth, Roland Veltkamp, Pankaj Sharma, Paul Guyler, Paul O'Mahony, Peter Wilkinson, Prabel Datta, Prasanna Aghoram, Rachel Marsh, Robert Luder, Sanjeevikumar Meenakishundaram, Santhosh Subramonian, Simon Leach, Sissi Ispoglou, Sreeman Andole, Timothy England, Aravindakshan Manoj, Frances Harrington, Habiba Rehman, Jane Sword, Julie Staals, Karim Mahawish, Kirsty Harkness, Louise Shaw, Michael McCormich, Nikola Sprigg, Syed Mansoor, Vinodh Krishnamurthy

**Affiliations:** From the Stroke Research Center (J.G.B., C.B., H.D., G.B., D.W., C.S., M.M.B., D.J.W.) and Lysholm Department of Neuroradiology and the Neuroradiological Academic Unit (T.A.Y., H.R.J.), Department of Brain Repair and Rehabilitation, UCL Queen Square Institute of Neurology; Department of Statistical Science (G.A.), Haemostasis Research Unit, Department of Haematology (H.C.), University College London, UK; Stroke Research Center, Department of Neurology (H.D.), Fujian Medical University Union Hospital, Fuzhou, China; Center for Clinical Brain Sciences, School of Clinical Sciences (R.A.-S.S.), University of Edinburgh; Liverpool Center for Cardiovascular Science (G.Y.H.L.), University of Liverpool and Liverpool Heart and Chest Hospital, UK; Aalborg Thrombosis Research Unit, Department of Clinical Medicine (G.Y.H.L.), Aalborg University, Denmark; Department of Molecular Neuroscience (H.H.), UCL Queen Square Institute of Neurology and the National Hospital for Neurology and Neurosurgery, London; and Institute of Neuroscience & Psychology (K.W.M.), University of Glasgow, Queen Elizabeth University Hospital, UK.

## Abstract

**Objective:**

To investigate whether enlarged perivascular spaces (PVS) within the basal ganglia or deep cerebral white matter are risk factors for intracranial hemorrhage in patients taking oral anticoagulants (OACs), independent of established clinical and radiologic risk factors, we conducted a post hoc analysis of Clinical Relevance of Microbleeds in Stroke (CROMIS-2) (atrial fibrillation [AF]), a prospective inception cohort study.

**Methods:**

Patients with atrial fibrillation and recent TIA or ischemic stroke underwent standardized MRI prior to starting OAC. We rated basal ganglia PVS (BGPVS) and centrum semiovale PVS (CSOPVS), cerebral microbleeds (CMBs), white matter hyperintensities, and lacunes. We dichotomized the PVS rating using a threshold of >10 PVS in the relevant region of either cerebral hemisphere. The primary outcome was symptomatic intracranial hemorrhage (sICH). We identified risk factors for sICH using Cox regression.

**Results:**

A total of 1,386 participants with available clinical and imaging variables were followed up for a mean of 2.34 years; 14 sICH occurred (11 intracerebral). In univariable analysis, diabetes, CMB presence, lacune presence, and >10 BGPVS, but not CSOPVS, were associated with sICH. In a multivariable model incorporating all variables with significant associations in univariable analysis, >10 BGPVS (hazard ratio [HR] 8.96, 95% [CI] 2.41–33.4, *p* = 0.001) and diabetes (HR 3.91, 95% CI 1.34–11.4) remained significant risk factors for sICH.

**Conclusion:**

Enlarged BGPVS might be a novel risk factor for OAC-related ICH. The strength of this association and potential use in predicting ICH in clinical practice should be investigated in larger cohorts.

Within the brain, the perivascular space (PVS) is the compartment bounded by the wall of penetrating cerebral blood vessels and the glia limitans, which might facilitate fluid circulation and clearance of soluble waste, including β-amyloid (Aβ), from brain parenchyma.^1–3^ In age and disease, PVS may enlarge and become MRI-visible, as fluid-filled structures most easily assessed within the basal ganglia and the centrum semiovale white matter. Cross-sectional studies of intracerebral hemorrhage (ICH) survivors have associated enlarged basal ganglia PVS (BGPVS) with deep ICH, increased white matter hyperintensity volume, and deep cerebral microhemorrhages, and enlarged centrum semiovale PVS (CSOPVS) with lobar ICH and cerebral amyloid angiopathy (CAA).^4–6^ In cognitively impaired patients, BGPVS are associated with hypertension, deep CMBs, and lacunes, and CSOPVS with lobar CMBS, cortical superficial siderosis, and Alzheimer disease.^7–9^

Together, these data suggest that BGPVS might be markers of deep perforator arteriopathy, and CSOPVS of Aβ pathology, including CAA. PVS might therefore also indicate increased ICH risk. A prospective study of patients with TIA or ischemic stroke found an association between >20 BGPVS in either hemisphere and incident ICH, though this was not statistically significant when adjusted for vascular risk factors.^10^ We investigated this question in patients with atrial fibrillation (AF) taking oral anticoagulants (OACs) after ischemic stroke or TIA. We hypothesized that BGPVS and CSOPVS would be associated with anticoagulant-related intracranial hemorrhage (OAC-ICH), independent of other markers of cerebral small vessel disease linked to OAC-ICH, notably cerebral microbleeds (CMBs) and white matter hyperintensities.^11,12^

## Methods

### Study design

We conducted a post hoc analysis of the Clinical Relevance of Microbleeds in Stroke (CROMIS-2) (AF) study, a multicenter prospective inception cohort study of the relationship between CMBs and anticoagulant-related symptomatic intracranial hemorrhage (sICH). The design, full description of the cohort, and primary results of this study have been published elsewhere.^11^ Briefly, we recruited adult patients with AF initiating oral anticoagulation after recent ischemic stroke or TIA from 79 hospitals in the United Kingdom and 1 in the Netherlands between August 2011 and July 2015. MRI was performed at baseline according to a standardized protocol, including axial T1- and T2-weighted, coronal fluid-attenuated inversion recovery (FLAIR), diffusion-weighted, and T2*-weighted gradient-recalled echo sequences. To reduce selection bias, imaging was performed after study enrollment, and we only enrolled participants whose responsible clinician had already decided to treat with an anticoagulant. We followed up participants for 24 months using multiple overlapping methods, including postal questionnaires, telephone interviews, and hospital episode statistics. The primary outcome was sICH, defined as brain imaging evidence of nontraumatic spontaneous intracranial hemorrhage with appropriate clinical symptoms.

### Neuroimaging analysis

We analyzed participants' MRIs for markers of cerebral small vessel disease according to STRIVE (Standards for Reporting Vascular Changes on Neuroimaging) definitions.^13^ Trained research fellows (J.G.B., D.W., G.B., H.D.) performed ratings blinded to the occurrence of sICH during follow-up, using validated rating scales where available. One of 2 raters (G.B., H.D.) rated PVS at the level of the basal ganglia and centrum semiovale separately, using a 5-level scale that assigns a score of 0 to no visible PVS, 1 to 1–10, 2 to 11–20, 3 to >21–40, and 4 to >40 PVS.^14^ We rated each hemisphere, unless prevented by the presence of a focal lesion, and used the higher of the 2 values. We rated CMBs using the Microbleed Anatomical Rating Scale^15^ and white matter hyperintensities using the Age-Related White Matter Changes (ARWMC) scale (D.W.).^16^ We identified and counted lacunes (G.B., H.D.).

Given the possibility that enlarged PVS might predominantly reflect cerebral atrophy, 1 of 2 raters (G.B., J.G.B.) rated each participant's imaging using the simplified Pasquier scale,^17^ which quantifies the global severity of cortical atrophy (GCA) on a 4-level scale (0: no atrophy, 1: sulcal widening, 2: gyral volume loss, 3: “knife-blade” atrophy). We used axial T1 or FLAIR images for rating if available, and inverted axial T2 images if not. When a significant focal lesion was present, we rated the nonlesioned hemisphere.

When more than 1 rater rated a marker, we assessed interrater agreement on a random sample using Cohen kappa, weighted for ordinal variables.

### Statistical analysis

We investigated the association between enlarged PVS and the hazard of sICH using Cox regression. As well as BGPVS and CSOPVS, we prespecified age, sex, clinical history of diabetes mellitus, and clinical history of hypertension as clinical independent variables and ARWMC score, CMBs, lacunes, and GCA as additional imaging independent variables. We chose not to include cortical superficial siderosis due to its very low prevalence in our study population. Using a predetermined threshold previously associated with the presence of other small vessel disease markers^14^ and incorporated into a validated composite small vessel disease score as representing moderate to severe PVS,^18^ we dichotomized PVS counts in our analysis as 0–10 or >10, equivalent to a PVS score of 0–1 and 2–4, respectively. We dichotomized CMBs and lacunes as present or absent. We included the ARWMC score as a continuous variable. As very few participants received a GCA rating of 3, we combined categories 2 and 3 to give a 3-level ordinal variable comprising no atrophy (0), minor atrophy (1), and moderate to severe atrophy (2–3). To reduce the risk of overfitting, we initially performed univariable analysis for each variable, and included only variables with an association at the 20% level in our final multivariable analysis. We checked the proportional hazards assumption using visual inspection of log-log plots of the log cumulative hazard against log time and through postestimation tests based on Schoenfeld residuals. We summarized our results graphically using plots of the Kaplan-Meier failure (1 − survival) function. Statistical analysis was performed using Stata version 15.0.

### Standard protocol approvals, registrations, and patient consents

The UK National Health Service Research Ethics Committee approved the CROMIS-2 study. Patients with capacity provided written informed consent. We obtained proxy written informed consent if patients lacked capacity to consent, following relevant local legislation.

### Data availability

We will share anonymized data on reasonable request, following consideration by the CROMIS-2 steering committee and execution of a data sharing agreement. Requests should be submitted to d.werring@ucl.ac.uk.

## Results

**Figure 1:**
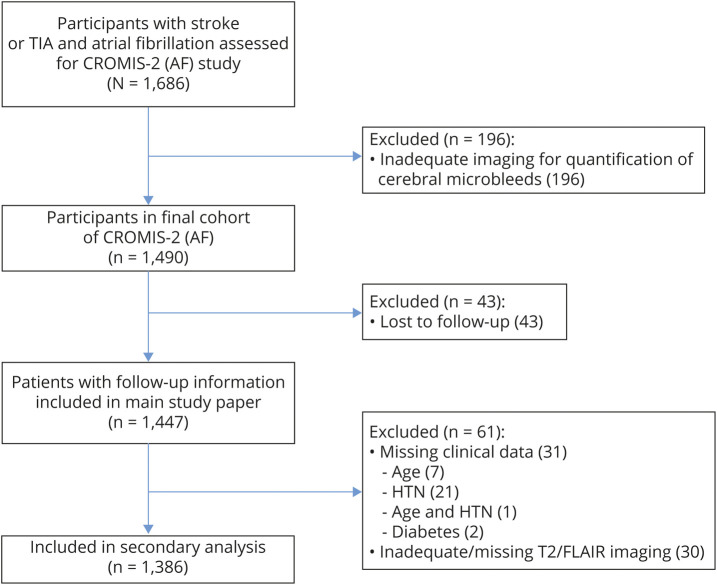
Study flowchart CROMIS-2 (AF) = Clinical Relevance of Microbleeds in Stroke (atrial fibrillation); FLAIR = fluid-attenuated inversion recovery; HTN = hypertension.

The primary analysis of the CROMIS-2 (AF) study included 1,447 participants who met the main study inclusion criteria and had follow-up data available. Of these, 1,386 participants (95.8%) had all additional variables of interest needed for this secondary analysis (figure 1). Participants excluded from our secondary analysis due to missing variables were more likely to be female and have hypertension. As other variables were comparable (table 1) and the overall proportion of missing data was very low, we performed a complete case analysis.

**Table 1:**
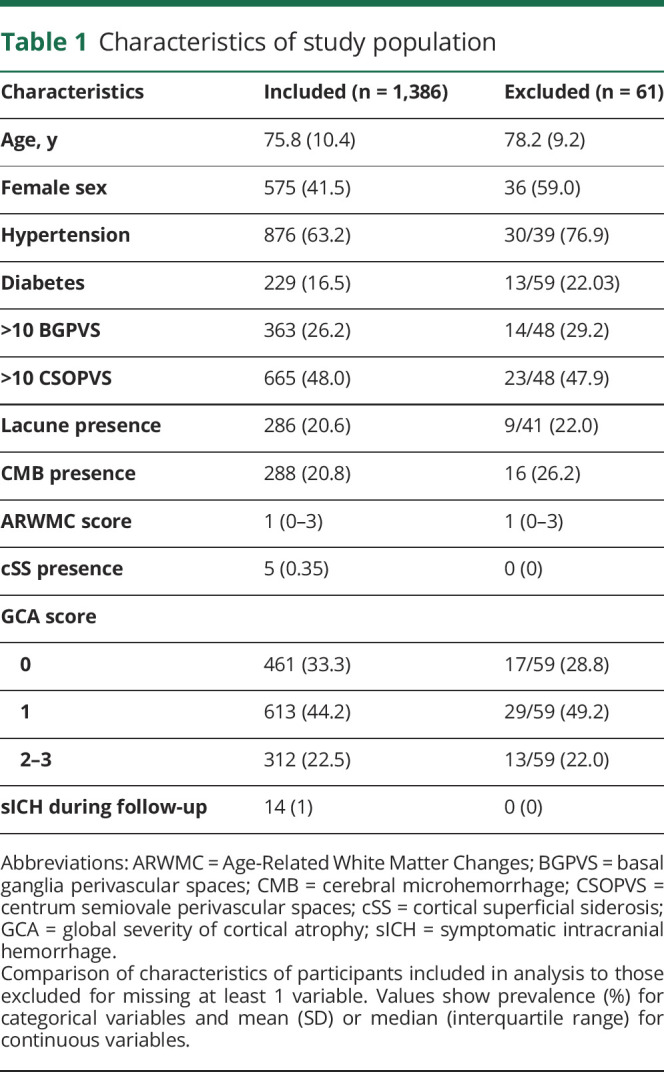
Characteristics of study population

During 3,251 participant-years of follow-up, 14 sICH occurred (11 intracerebral, 2 subdural, 1 subarachnoid). The median time from anticoagulation initiation to sICH was 272 days (interquartile range 211–657). Of the 10 intracerebral hemorrhages for which data on location were available, 2 were deep, 4 were infratentorial, and 4 were lobar. Table 2 summarizes the clinical and radiologic characteristics of participants with and without sICH during follow-up. Interrater agreement for dichotomized PVS score was excellent (k = 0.82) within the basal ganglia and substantial (k = 0.80) within the centrum semiovale (n = 50). Agreement for GCA rating was moderate (k = 0.53; n = 100), comparable to that in other existing literature.^19^ We observed a weak correlation between GCA and BGPVS grade (Spearman ρ 0.17, 95% confidence interval [CI] 0.12–0.22), but not CSOPVS grade (*r* = 0.04, 95% CI −0.01 to 0.01).

**Table 2:**
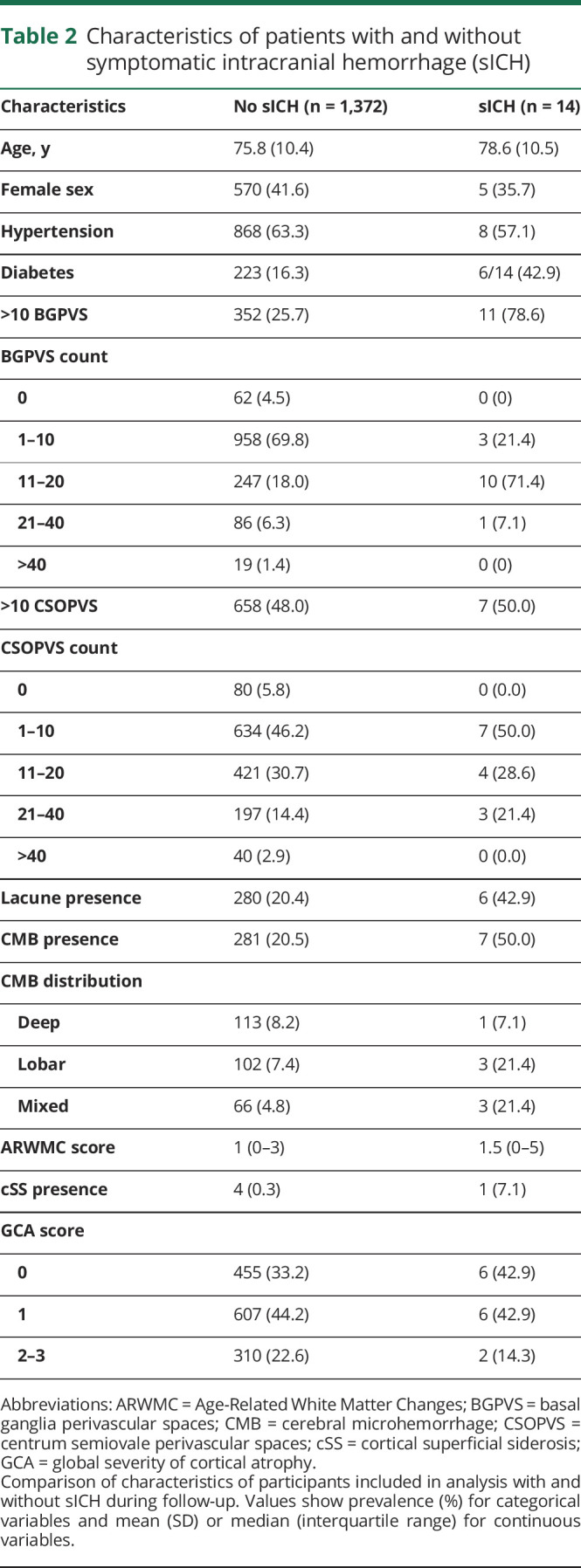
Characteristics of patients with and without symptomatic intracranial hemorrhage (sICH)

**Figure 2:**
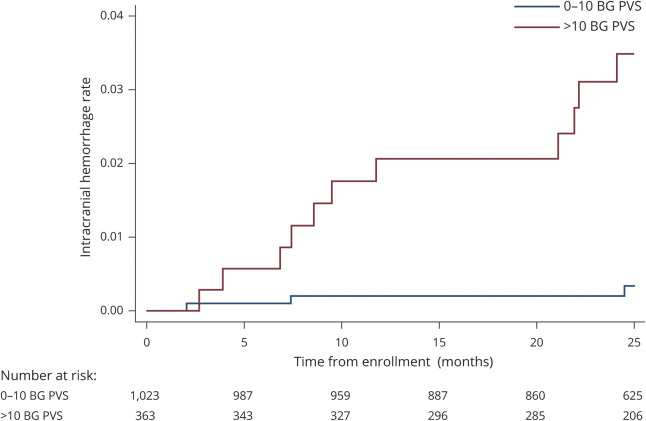
Cumulative probability of symptomatic intracranial hemorrhage by basal ganglia (BG) perivascular space (PVS) rating

Univariable Cox regression showed associations between BGPVS, diabetes, lacune presence, and CMB presence and sICH (table 3). We found no evidence of an association between CSOPVS and sICH. In a multivariable model incorporating diabetes, BGPVS, CMB presence, and lacune presence, we found strong evidence of an association with sICH for enlarged BGPVS and diabetes, and weak evidence of an association for CMB presence. No evidence of an association was found for lacune presence. Figure 2 shows the cumulative incidence of sICH during study follow-up for participants with 0–10 and >10 BGPVS. The absolute rate of sICH in participants with >10 BGPVS was 1.38/100 participant-years (95% CI 0.69–2.47), compared to 0.12/100 participant-years (95% CI 0.025–0.36) in participants with 0–10 BGPVS.

**Table 3:**
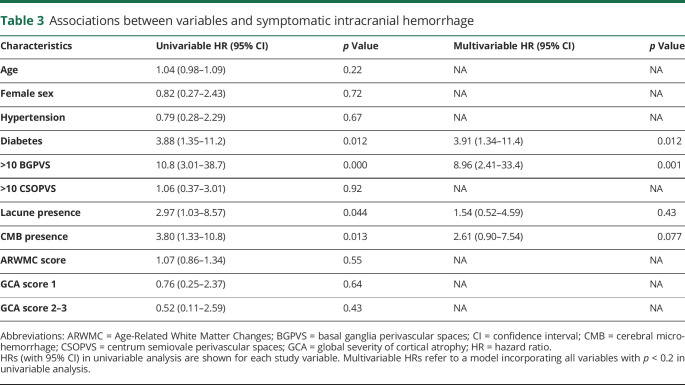
Associations between variables and symptomatic intracranial hemorrhage

Given the number of variables included in our multivariable model relative to the number of outcome events, we performed additional sensitivity analyses, testing each combination of BGPVS presence and diabetes, CMB presence, or lacune presence individually (table 4)*.* The result of each analysis was similar to that of the main multivariable model. We also undertook a sensitivity analysis to investigate the effect of dichotomizing PVS as 0–20 or >20, rather than 0–10 or >10. Using this threshold, we did not find an association between higher BGPVS or CSOPVS counts and sICH (BGPVS: hazard ratio [HR] 1.0, 95% CI 0.13–7.5; CSOPVS: HR 1.3, 95% CI 0.36–4.6); however, few participants in our study had counts >20 in either location (table 2), and CIs for both estimates were wide.

**Table 4:**
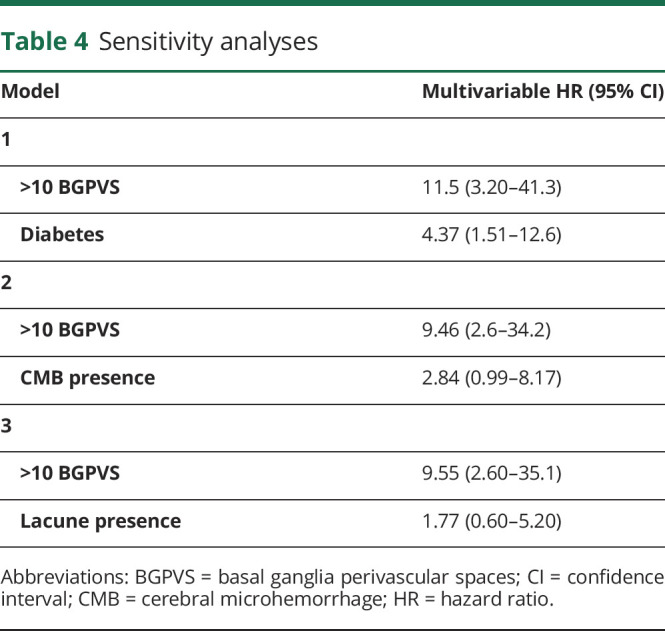
Sensitivity analyses

For each model, visual inspection of log-log plots suggested no violation of the proportional hazards assumption. Although postestimation tests provided some evidence that the assumption was violated for hypertension (*p* = 0.037), the estimate of the hazard ratio for sICH for hypertension provided no evidence for an association, and hypertension was not included in our multivariable model.

## Discussion

Our main finding is an association between enlarged PVS within the basal ganglia, but not centrum semiovale, and OAC-ICH, independent of major vascular risk factors and other markers of cerebral small vessel disease. The estimate and 95% CI of the HR was consistent with a clinically meaningful association, and was highly statistically significant, though it lacked precision. Although preliminary, our finding raises the possibility that enlarged BGPVS might be a clinically relevant marker of OAC-ICH risk. Other studies have provided supportive observational evidence that incorporating small vessel disease markers, specifically CMB presence and WMH severity, can improve the performance of clinical risk scores for ICH,^11,12^ and the current analysis suggests that incorporating BGPVS status into these scores might usefully be investigated. An advantage of BGPVS status as a marker might be that it can be quantified on axial T2 imaging, a routine component of nearly all clinical brain MRI.

Our findings add to the evidence linking enlarged PVS to cerebrovascular disease, but why PVS enlargement might occur in the setting of hemorrhage-prone cerebral small vessel disease remains unclear. PVS enlargement might reflect extravasation of fluid across damaged small vessel walls, possibly compounded by recruitment of inflammatory cells to the PVS, where they might promote further loss of blood–brain barrier integrity and impair perivascular fluid transport.^20^ In CAA, perivascular aggregation of Aβ may also impair drainage,^21^ but this is less likely to mediate the association between BGPVS and ICH we observed. It is possible that more advanced cerebral small vessel disease might lead directly to PVS enlargement through ischemic brain atrophy. We consider this less likely, as we corrected for a measure of overall cerebral atrophy, and observed an association that was independent of other cerebral small vessel disease markers, more consistent with BGPVS enlargement being a sensitive marker or early feature of cerebral small vessel disease. Finally, the association between BGPVS and ICH might be mediated by a shared underlying mechanism. For example, arterial stiffening has recently been associated with BGPVS,^22^ adjusted for major vascular risk factors, and also with deep ICH cross-sectionally and new CMB formation prospectively.^23,24^ By reducing damping of the cardiac impulse and increasing transmission of pulsatile force to small cerebral arteries,^25^ arterial stiffening might increase ICH risk, and promote PVS formation by altering small vessel pulsatility, thought to be a key driver of perivascular fluid transport.^26,27^

Unexpectedly, we did not find an association between CSOPVS and sICH, despite evidence linking CSOPVS to CAA. We considered whether CSOPVS might simply be more difficult to measure reliably than BGPVS, leading to increased statistical noise and difficulty in detecting any associations that do exist, but our excellent interrater reliability for both BGPVS and CSOPVS argues against this. More likely explanations include the low specificity of the association between CSOPVS and CAA, as CSOPVS enlargement also occurs in Alzheimer disease without clinical CAA, and the low proportion of our study population (3%) who met modified Boston criteria for CAA, probably because such patients are not generally viewed as eligible for anticoagulation. Finally, differences in regional vascular anatomy might contribute. Whereas the hemispheric white matter is supplied by penetrating branches of distal cortical arteries, the basal region of the brain is supplied by small perforating arteries that arise directly from large cerebral arteries. These vessels are therefore exposed to higher peak blood pressures,^28^ and so to the effects of systemic hypertension and, potentially, arterial stiffening. BGPVS enlargement might therefore be a more sensitive marker of these processes than CSOPVS.

As a secondary analysis of CROMIS-2 (AF), our study has methodologic strengths: this was a large, multicenter prospective inception cohort study, recruiting a population similar in age, prevalence of vascular risk factors, and stroke severity to the overall case mix of UK stroke units.^29^ We obtained a >97% follow-up rate, and sICH events were adjudicated centrally without knowledge of CMB or PVS status. The MRI protocol was standardized between sites, and imaging markers were rated blinded to sICH during follow-up. We obtained substantial or excellent interrater agreement for key small vessel disease markers.

Our study has limitations. Most importantly, as a post hoc analysis, our results should be considered hypothesis-generating. The need for verification of our findings in an independent cohort is emphasized by the low number of outcome events observed, although we attempted to limit overfitting of our multivariable regression model by using a variable-selection procedure, and undertook additional sensitivity analyses. We included a clinical history of hypertension as an independent variable in our analysis, but we lacked data on BP control prior to or during the study, as well as details of anticoagulation control intensity for warfarin-treated patients, and adherence to anticoagulation for all participants. As eligibility for anticoagulation was an inclusion criterion for the study, our cohort might not be fully representative of the overall population of patients with AF in whom anticoagulation is considered, and although study-specific imaging was performed after the decision to treat with anticoagulation was made, we cannot exclude the possibility that patient selection to our study may have been influenced by the results of imaging already performed as part of clinical care. The generalizability of our result may be further affected by our predominantly (95%) Caucasian study population, and the low proportion of patients taking direct oral anticoagulants (37%), which are increasingly preferred to warfarin in clinical practice due to their lower risk of ICH. Of the 14 ICH events observed in our study, 12 were in warfarin-treated patients.

As well as validating our findings, further study in a large independent cohort is needed to allow precise estimation of the strength of the association between BGPVS and OAC-ICH, and investigation of its association with BGPVS count or score, rather than the dichotomized rating used in this study. We failed to observe an association between >20 BGPVS and sICH, in contrast to a previous study,^10^ which we attribute to the low prevalence of higher PVS counts in our study and the small number of outcome events observed. However, the relationship between BGPVS count and sICH might also be nonlinear, showing a threshold effect, and larger, better-powered studies might clarify this. Such studies might also investigate the relationship between PVS and different locations of sICH (for example, lobar vs deep/infratentorial intracerebral haemorrhage, or intracerebral vs subdural or subarachnoid hemorrhage), which have different biological mechanisms.

The clinical importance of our finding will depend on whether adding BGPVS status to existing ICH risk models can improve their performance, which we chose not to investigate in our cohort due to the risk of overfitting, and clarification of whether BGPVS are also associated with ischemic stroke, and the strength of this association, if present, relative to that with ICH. This information is needed to establish whether BGPVS status should influence the selection of pharmacologic and nonpharmacologic treatments for stroke prevention in patients with AF in clinical practice. Large-scale global collaboration between cohort studies of OAC-related ICH, such as the Microbleeds International Collaborative Network,^30^ might provide a means by which to investigate these unanswered questions.

## Glossary

Aβ: β-amyloid
ARWMC: Age-Related White Matter Changes
AF: atrial fibrillation
BGPVS: basal ganglia perivascular spaces
CAA: cerebral amyloid angiopathy
CI: confidence interval
CMB: cerebral microbleed
CROMIS-2: Clinical Relevance of Microbleeds in Stroke
CSOPVS: centrum semiovale perivascular spaces
FLAIR: fluid-attenuated inversion recovery
GCA: global severity of cortical atrophy
HR: hazard ratio
ICH: intracerebral hemorrhage
OAC: oral anticoagulant
PVS: perivascular space
sICH: symptomatic intracranial hemorrhage
